# An Exonuclease I-Based Quencher-Free Fluorescent Method Using DNA Hairpin Probes for Rapid Detection of MicroRNA

**DOI:** 10.3390/s17040760

**Published:** 2017-04-03

**Authors:** Changbei Ma, Haisheng Liu, Kefeng Wu, Mingjian Chen, Liyang Zheng, Jun Wang

**Affiliations:** State Key Laboratory of Medical Genetics & School of Life Sciences, Central South University, Changsha 410013, China; 15675888046@163.com (H.L.); Kefeng@csu.edu.cn (K.W.); macblab@163.com (M.C.); zg_zly@126.com (L.Z.)

**Keywords:** microRNA, quencher-free, exonuclease I, 2-aminopurine, hairpin probe

## Abstract

MicroRNAs (miRNAs) act as biomarkers for the diagnosis of a variety of cancers. Since the currently used methods for miRNA detection have limitations, simple, sensitive, and cost-effective methods for the detection of miRNA are required. This work demonstrates a facile, quencher-free, fluorescence-based analytical method for cost-effective and sensitive detection of miRNA using a super 2-aminopurine (2-AP)-labeled hairpin probe (HP) and exonuclease I activity. Specifically, the fluorescence of 2-AP is strongly quenched when it is incorporated within DNA. In the presence of a target miRNA, HP attains an open conformation by hybridizing with the target miRNA to form a double-stranded structure with a protruding 3′-terminus. Next, the digestion of the protruding 3′-terminus is triggered by exonuclease I, during which 2-AP is released free in solution from the DNA, thereby increasing fluorescence. This method is highly sensitive, with a detection limit of 0.5 nM—10 times lower than a previously reported quencher-free fluorescence method. Furthermore, this method has potential applications in clinical diagnosis and biomedical research.

## 1. Introduction

MicroRNAs (miRNAs) are a class of small (19–23 nucleotides long), single-stranded, endogenous, non-coding RNAs that commonly exist in plant, animal, and virus genomes, and were first discovered in the 1990s [1,2,3]. It has been demonstrated that miRNAs play key regulatory roles in a series of critical biological processes, such as metabolism, differentiation, apoptosis, and immunological response [4,5]. Since abnormal miRNA expression is associated with a variety of cancers, such as cancers of the breast, prostate, liver, lung, and colon, miRNAs have drawn considerable attention as putative biomarkers and therapeutic targets for cancer treatment [6,7,8]. Therefore, devising rapid and sensitive methods for the detection of miRNAs is important.

Several traditional mainstream techniques for miRNA detection and quantitation are Northern blotting, quantitative reverse transcription polymerase chain reaction (qRT-PCR), and microarray analysis; however, each of these methods have limitations which hinder their wide and convenient application [9,10,11]. Northern blotting is widely used in miRNA detection and quantitation, although it is time-consuming and requires the use of radiolabels and large sample size [12,13]. qRT-PCR is a highly sensitive method; however, the methodology requires tedious preparation and highly-skilled personnel [12]. Microarray is a high-throughput method, but the analysis platform is not suitable for quantitating rare miRNAs, owing to its low sensitivity [13]. Hence, pursuits for alternative analytical methods that circumvent the above limitations have received increasing attention. Recently, a variety of device-based analytical strategies and methods-based approaches have been developed to quantitatively analyze miRNAs. Device-based analytical strategies mainly include electrochemical sensors and MEMS [14,15]. Methods-based approaches mainly include fluorescence, colorimetric, and luminescence assays [16,17,18,19,20,21,22,23,24,25,26,27,28]. Among these, the fluorometric method is an excellent choice for the quantitative detection of miRNAs due to its high sensitivity and convenience of operation. Santangelo and co-workers have reported a facile method for the detection of miRNA using dual fluorescence resonance energy transfer (FRET) molecular beacons [29]. Ryoo et al. have developed a method to detect miRNA based on nano graphene oxide (PANGO—peptide nucleic acid and nano graphene oxide) [30]. Yin et al. have developed a novel method to detect miRNAs based on duplex-specific nuclease signal amplification [31]. Zhang et al. have designed a strategy for the detection of miRNAs by a catalyst–oligomer-mediated enzymatic amplification-based fluorescence biosensor, which is a highly-sensitive method [32]. Although these strategies are usually rapid and highly sensitive, they still utilize expensive dual labeled probes or tedious preparations. Therefore, developing a facile, sensitive, and low-cost analytical method to detect miRNAs is still an open challenge.

2-Aminopurine (2-AP)—whose fluorescence is strongly quenched when incorporated within DNA—has been used as a fluorescent analogue of adenine for many years [33]. 2-AP is often used as a quencher-free fluorescent probe for the study of protein–DNA interactions and DNA flipping [34,35]. The quenching of 2-AP fluorescence results from its stacking interaction with adjacent bases of DNA without the involvement of any extra quenchers [36,37]. Exonuclease I (exo I) belongs to the exonuclease family that can degrade single-stranded DNA in a 3′→5′ direction and release deoxyribonucleoside 5′-monophosphates in a step-wise manner. Here, we developed a facile quencher-free fluorescence-based analytical strategy for sensitive and low-cost detection of miRNAs using a super 2-AP-labeled hairpin probe (HP) and exo I activity. By taking advantage of the super quencher-free fluorescence-labeled 2-AP probe, we provide a facile, low-cost, and sensitive analytical method for quantitating miRNA [38,39].

## 2. Materials and Methods

### 2.1. Reagents

miRNA-21 (5′-UAGCUUAUCAGACUGAUGUUGA-3′), miR-141 (5′-UAACAC UGUCUGGUAAAGAUGG-3′), and let-7d (5′-AGAGGUAGUAGGUUGCAUAGUU-3′) synthesized by TaKaRa Biotechnology Co., Ltd. (Dalian, China) were purified by high-performance liquid chromatography (HPLC). Hairpin probe (HP: 5′-GCG TCG TCA ACA TCA GTC TGA TAA GCT ACG /2-amp/CGC-3′), which were purified by HLPC, were obtained from Sangon Biotechnology Co., Ltd. (Shanghai, China). Exonuclease I, 10× exonuclease I buffer (670 mM Glycine-KOH, 10 mM Dithiothreitol (DTT), 67 mM MgCl_2_, pH 9.5), recombinant RNase inhibitor (RRI), and RNase-free water were obtained from TaKaRa Biotechnology Co., Ltd. (Dalian, China). Human serum samples were collected from the third Xiangya hospital of Central South University (Changsha, China). All the reagents were used without further purification. All the nucleic acids used in this study were dissolved in RNase-free water and stored at −20 °C until further use.

### 2.2. Fluorescence Measurement

All fluorescence measurements were carried out on an F2700 (Hitachi, Japan) with excitation at 310 nm and emission at 365 nm for 2-AP. The excitation slits and emission slits were both set at 5.0 nm.

### 2.3. Detection of miRNA

Two samples were prepared: sample A contained only 200 nM HP, whereas sample B contained 200 nM HP and 200 nM miRNA-21. Next, 20 U RRI and 20 U/mL exo I were added to samples A and B, respectively. Assays were carried out in 1× exonuclease I buffer (67 mM Glycine-KOH, 1 mM DTT, 6 mM MgCl_2_, pH 9.5). RNase-free water was used in all the reactions. Thereafter, samples A and B were incubated at 37 °C for approximately 30 min before taking measurements.

### 2.4. Optimization of Analysis Conditions

Certain critical reactions were optimized with respect to the time of reaction and the concentrations of HP and exo I that were used. The HP concentration range used was 50 to 400 μM, and the exo I concentration range was 3 to 40 U/mL; the reaction time range was 5 to 40 min.

### 2.5. Quantitative Analysis of miRNA

HP (200 nM), 20 U RRI, 20 U/mL exo I, and different amounts of miRNA-21 were assembled in 100 μL of 1× exo I buffer and incubated at 37 °C for about 30 min before taking measurements. miRNA-21 concentration in the reaction varied from 0 to 200 nM.

### 2.6. Application of the Method in Biological Assays

Enzymes and proteins were removed from human serum by calefaction, following which the serum was diluted 1:10 with phosphate-buffered saline (PBS) buffer, and then spiked with miRNA at three concentrations (50, 100, and 200 nM miRNA-21) and analyzed.

## 3. Results and Discussion

### 3.1. Designing the Strategy

**Figure 1:**
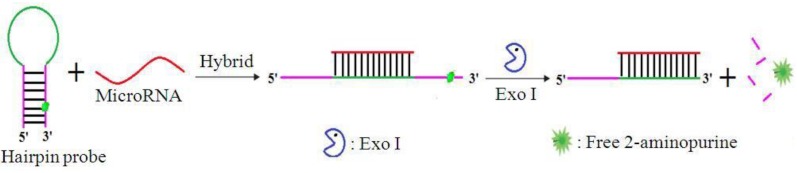
Schematic showing the principle of the quencher-free fluorescence-based method for the detection of microRNA. Exo I: Exonuclease I.

The working principle of the quencher-free fluorescence-based method for miRNA detection is illustrated in Figure 1. The reaction system consists of HP, exo I, and miRNA. Exo I specifically degrades single-stranded DNA (ssDNA) in a 3′→5′ direction, and has no activity on double-stranded DNA (dsDNA). The HP has two domains: a 2-AP labeled stem without overhanging nucleotides, which protects the HP from exo I digestion and a loop sequence which is complementary to the target miRNA. In the absence of the target miRNA, 2-AP is incorporated within HP, and its fluorescence is quenched. Once the target miRNA hybridizes with the HP, the latter attains an open conformation and forms a double-stranded structure with a protruding 3′-terminus, which triggers the digestion of the protruding 3′-terminus by exo I. During the digestion, 2-AP is separated from the DNA strands and released free in solution, thereby increasing fluorescence. Thus, the fluorescence intensity observed at 365 nm is directly proportional to the concentration of miRNA present, allowing quantitative detection of the target miRNA.

### 3.2. Validation of miRNA Detection with the Method

**Figure 2:**
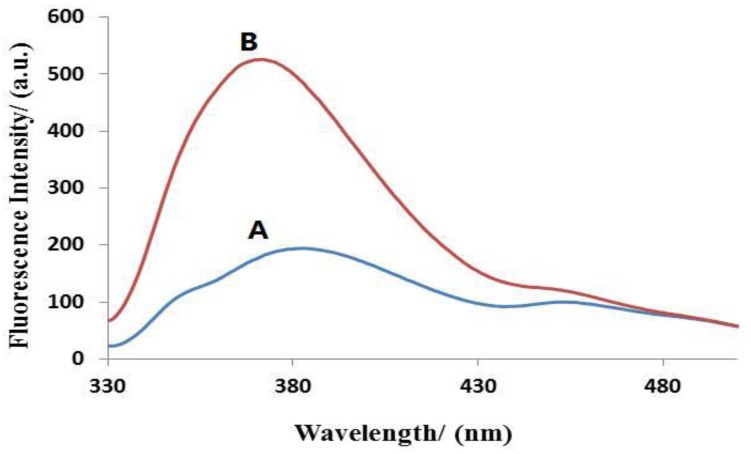
Validation of microRNA (miRNA) detection by the proposed method. Fluorescence emission spectra obtained when (**A**) no target miRNA and (**B**) 200 nM target miRNA were used in the reaction.

We evaluated the feasibility of the proposed method for miRNA detection by using two samples. Figure 2 depicts the fluorescence emission spectra in the presence and absence of target miRNA. The fluorescence intensity was weak in the absence of target miRNA (curve A). When the target miRNA was added to reaction solution, an increase in fluorescence intensity was observed (curve B), which confirmed that our assay can actually detect miRNAs.

### 3.3. Optimization of Experimental Conditions

**Figure 3:**
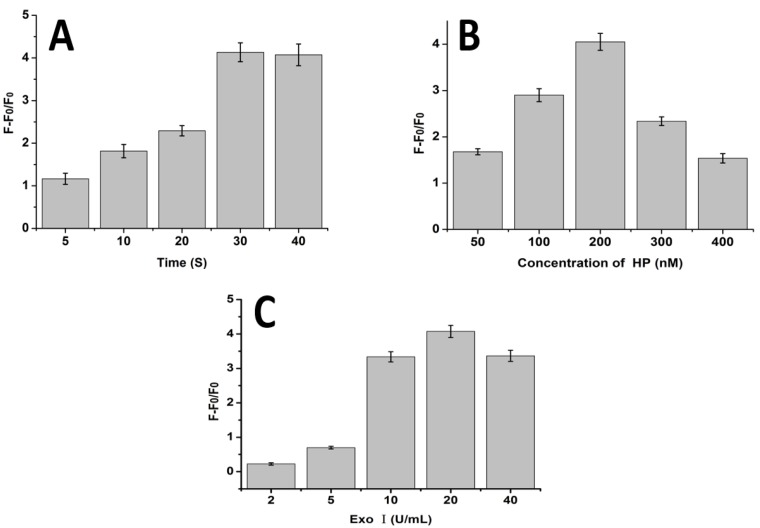
Optimization of miRNA detection conditions. (**A**) Bar graph showing the relationship between reaction time and (F0-F)/F0. Each sample contained 200 nM HP, 200 nM miRNA, and 20 U/mL exo I. (**B**) Bar graph showing the relationship between the concentration of HP and (F0-F)/F0. In each sample, 200 nM miRNA and 20 U/mL exo I were present. (**C**) Bar graph showing the relationship between the concentration of exo I and (F0-F)/F0. In each sample, 200 nM HP and 200 nM miRNA were present. Error bars were estimated from three replicate measurements.

Since reaction time and concentrations of HP and exo I can influence the performance of the proposed method, we first optimized these conditions to obtain the best result. After a series of experiments, we found that 30 min reaction time, 200 nM HP, and 20 U/mL exo I were the optimal reaction conditions (Figure 3). Accordingly, these conditions were used in the subsequent experiments.

### 3.4. Quantitative Analysis of miRNA

**Figure 4:**
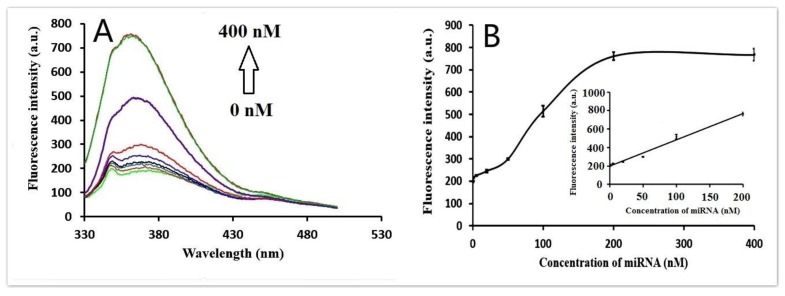
(**A**) Fluorescence emission spectra in the presence of increasing amounts of miRNA (0, 0.5, 1, 5, 20, 50, 100, 200, 400 nM). (**B**) Plot showing changes in fluorescence intensity with miRNA concentration. Inset: graph showing linear relationship of fluorescence intensity with miRNA concentration at low concentration range; 200 nM HP and 20 U/mL exo I were used. Error bars were estimated from three replicate measurements.

Various concentrations of target miRNA (miRNA-21) (0, 0.5, 1, 5, 20, 50, 100, 200, 400 nM) were added to the reaction sample to evaluate the sensitivity of the proposed method under the optimized conditions. The corresponding fluorescence intensity at 365 nm dynamically increased with increase in target miRNA concentration (Figure 4A). Figure 4B shows the relationship between fluorescence intensity and the concentration of target miRNA. The linear regression equation was F = 2.8402C + 198.96 (C, nM) with an R^2^ of 0.996, where F was the fluorescence intensity at 365 nm and C was the target miRNA concentration. The limit of detection (LOD) of the proposed strategy was estimated to be 0.5 nM, which was 10 times lower than a previously reported quencher-free fluorescence method (5 nM) [40].

### 3.5. Selectivity of the miRNA Detection Assay

**Figure 5:**
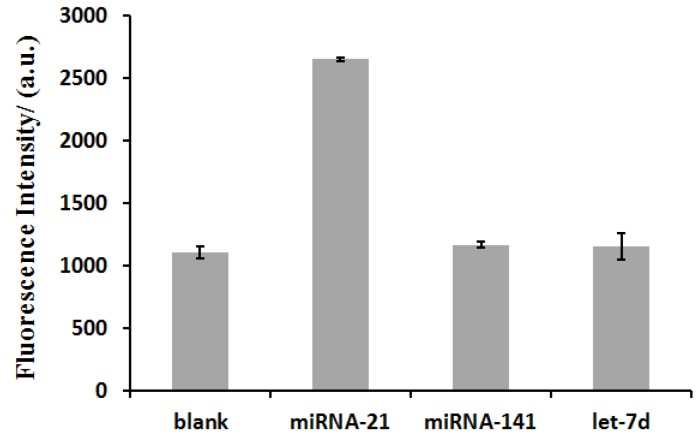
The proposed method is highly selective for target miRNA detection. Respectively 800 nM of miRNA-141 and let-7d were used in the presence of 200 nM HP and 20 U/mL exo I. The concentration of miRNA-21 was 200 nM. “Blank” is the sample without miRNA. Error bars were estimated from three replicate measurements.

The selectivity of this miRNA biosensor was investigated by using 800 nM of two mismatched miRNA sequences—namely, miRNA-141 and let-7d. As shown in Figure 5, none of these mismatched miRNA sequences could trigger a significant increase in fluorescence intensity except the target miRNA, miRNA-21 (200 nM). These results underline the ability of our method to distinguish between perfectly matched target miRNAs and mismatched miRNAs, suggesting that it can be used for miRNA detection.

### 3.6. Application of the Method in Biological Assays

A literature survey revealed that miRNA-21 concentration in human serum is about 35 fM, which is lower than the detection limit of the proposed method [41]. To demonstrate the feasibility of the approach in biological matrices, the human serum samples were spiked with miRNA at three concentrations (50, 100, and 200 nM miRNA-21), and analyzed. The analytical results are shown in Table 1. The fluorescence recovery rates for the different concentrations of target miRNA were 89% for 50 nM, 104.4% for 100 nM, and 99.4% for 200 nM. This indicates that the method may be used for analysis of miRNA in real biological samples. 

## 4. Conclusions

In summary, we have successfully demonstrated a user-friendly, quencher-free fluorescence-based method for sensitive miRNA detection based on an exonuclease I-catalyzed DNA degradation reaction. Firstly, the LOD of the quencher-free fluorescence method was estimated to be 0.5 nM, which is 10 times lower than a previously reported quencher-free fluorescence method. Secondly, by taking advantage of the quencher-free fluorescent 2-AP, we developed a low-cost and sensitive assay. Thirdly, the total reaction time in the proposed method is only 30 min, which is much shorter than those in most existing methods. We envision that our quencher-free fluorescence-based strategy for quantitative analysis of miRNA may have potential applications in clinical diagnosis and biomedical research.

## Figures and Tables

**Table 1:** Recovery of miRNA in diluted human serum using the proposed method.

Sample	Initial Concentration of miRNA-21 (nM)	Concentration of Recovered miRNA-21 (nM)	Recovery (%)
1	50	44.5	89
2	100	104.4	104.4
3	200	198.8	99.4
